# Using Artificial Intelligence Resources in Dialysis and Kidney Transplant Patients: A Literature Review

**DOI:** 10.1155/2020/9867872

**Published:** 2020-06-10

**Authors:** Alexandru Burlacu, Adrian Iftene, Daniel Jugrin, Iolanda Valentina Popa, Paula Madalina Lupu, Cristiana Vlad, Adrian Covic

**Affiliations:** ^1^Department of Interventional Cardiology-Cardiovascular Diseases Institute, Iasi, Romania; ^2^“Grigore T. Popa” University of Medicine, Iasi, Romania; ^3^Faculty of Computer Science, “Alexandru Ioan Cuza” University of Iasi, Romania; ^4^Center for Studies and Interreligious and Intercultural Dialogue, University of Bucharest, Romania; ^5^Institute of Gastroenterology and Hepatology, Iasi, Romania; ^6^Department of Internal Medicine-Nephrology, Iasi, Romania; ^7^Nephrology Clinic, Dialysis and Renal Transplant Center-‘C.I. Parhon' University Hospital, Iasi, Romania; ^8^The Academy of Romanian Scientists (AOSR), Romania

## Abstract

**Background:**

The purpose of this review is to depict current research and impact of artificial intelligence/machine learning (AI/ML) algorithms on dialysis and kidney transplantation. Published studies were presented from two points of view: What medical aspects were covered? What AI/ML algorithms have been used?

**Methods:**

We searched four electronic databases or studies that used AI/ML in hemodialysis (HD), peritoneal dialysis (PD), and kidney transplantation (KT). Sixty-nine studies were split into three categories: AI/ML and HD, PD, and KT, respectively. We identified 43 trials in the first group, 8 in the second, and 18 in the third. Then, studies were classified according to the type of algorithm.

**Results:**

AI and HD trials covered: (a) dialysis service management, (b) dialysis procedure, (c) anemia management, (d) hormonal/dietary issues, and (e) arteriovenous fistula assessment. PD studies were divided into (a) peritoneal technique issues, (b) infections, and (c) cardiovascular event prediction. AI in transplantation studies were allocated into (a) management systems (ML used as pretransplant organ-matching tools), (b) predicting graft rejection, (c) tacrolimus therapy modulation, and (d) dietary issues.

**Conclusions:**

Although guidelines are reluctant to recommend AI implementation in daily practice, there is plenty of evidence that AI/ML algorithms can predict better than nephrologists: volumes, Kt/V, and hypotension or cardiovascular events during dialysis. Altogether, these trials report a robust impact of AI/ML on quality of life and survival in G5D/T patients. In the coming years, one would probably witness the emergence of AI/ML devices that facilitate the management of dialysis patients, thus increasing the quality of life and survival.

## 1. Introduction

Artificial intelligence (AI) solutions are currently present in all medical and nonmedical fields. New algorithms have evolved to handle complex medical situations where the medical community has reached a plateau [1]. Medical registries received machine learning (ML) solutions for a better prediction of events that beat human accuracy [2]. Since AI/ML “*…have the potential to adapt and optimize device performance in real-time to continuously improve health care for patients…*,” the US Food and Drug Administration released this year a regulatory framework for modifications in the AI/ML-based software as a medical device [3].

The same board has approved in the last year at least 15 AI/deep learning platforms involved in the medical field (e.g., for atrial fibrillation detection, CT brain bleed diagnosis, coronary calcium scoring, paramedic stroke diagnosis, or breast density via mammography) [4].

In the last 15 years, numerous issues and complications generated by the end-stage renal disease requiring dialysis [5] (chronic kidney disease (CKD) stage G5D), by the technique of dialysis itself or by kidney transplantation (CKD stage G5T) [6], received incipient inputs from AI algorithms. However, the implementation of AI solutions in the dialysis field is still at the beginning. None of the successes reported above and approved by the FDA could be found in renal replacement therapy. Given the significant influence on healthcare in medical image processing, smart robotics in surgery, or Apple's watch impact on atrial fibrillation detection, the nephrology community recently raised two questions: “*Can this success be exported to dialysis? Is it possible to design and develop smart dialysis devices?*” [1].

If we are to be realistic, most of the end-stage renal disease (ESRD) patients (those who cannot benefit from kidney transplantation) are reliant on technology: currently, without a dialysis machine (the so-called “*artificial kidney*”), it is almost impossible to stay alive. It is evident that from the desire to create safer and more physiological devices, the nephrology community and the patients could benefit from AI/ML solutions powering actual machines or other improved versions. In other words, enhancing the functionality of the “*artificial kidney*” with “*artificial intelligence*” could constitute the next significant step toward better management of G5D patients.

In this regard, in 2019 was published probably one of the most suggestive and advanced examples: a multiple-endpoint model was developed to predict session-specific Kt/V, fluid volume removal, heart rate, and blood pressure based on dialysis-patient characteristics, historic hemodynamic responses, and dialysis-related prescriptions [7]. This research opens the door to other AI studies in ESRD patients in which an ML-powered machine would continuously and autonomously change its parameters (temperature, dialysate electrolyte compositions, duration, and ultrafiltration rates) in order to avoid one of the most vexing situations in dialysis (e.g., hypotension). Also, in this future framework, nephrology would indeed be “*personalized medicine*,” since a dialysis session would not be the same.

Our review's purpose is to depict the current research and impact of AI/ML algorithms on renal replacement therapy (hemo-, peritoneal dialysis, and kidney transplantation). We intend to summarize all studies published on this topic, presenting data from two points of view: (a) what medical aspects were covered? (b) What AI/ML algorithms have been used?.

## 2. Materials and Methods

**Figure 1:**
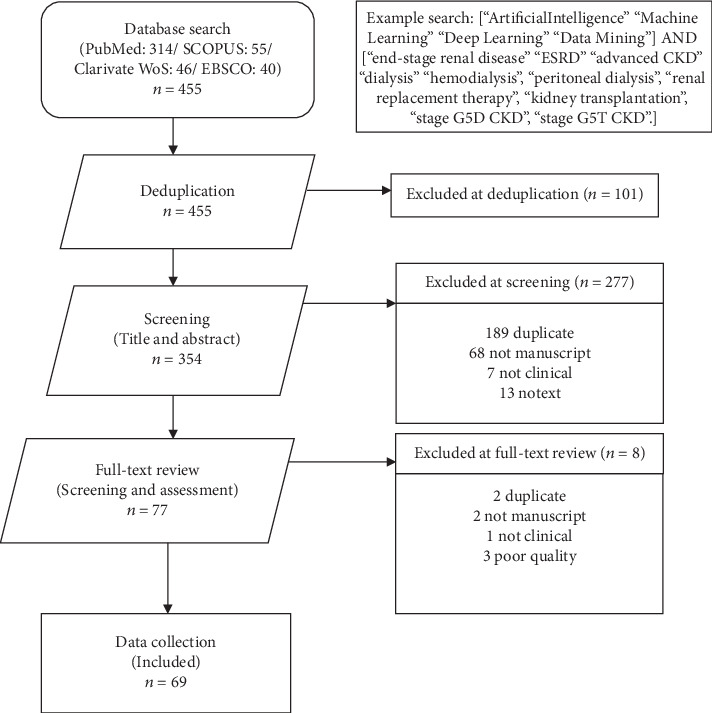
PRISMA flowchart for including articles in our study.

Our review adheres as strictly as possible to the PRISMA guidelines. The overall workflow is shown in Figure 1 and described below.

### 2.1. Search Strategy

We searched the electronic databases of PubMed, SCOPUS, Web of Science, and EBSCO from its earliest date until August 2019 for studies using AI/ML algorithms in dialysis and kidney transplantation. The terms used for searching were “*artificial intelligence*”, “*machine learning*”, “*deep learning*”, “*data mining*”, AND, “*end-stage renal disease*”, “*ESRD*”, “*advanced CKD*”, “*dialysis*”, “*hemodialysis*”, “*peritoneal dialysis*”, “*renal replacement therapy*”, “*kidney transplantation*”, “*stage G5D CKD*”, “*stage G5T CKD*”. The reference sections of relevant articles were also searched manually for additional publications (Figure 1). RCTs and observational studies, including prospective or retrospective cohort studies, reviews, meta-analyses, and guidelines were included if referring to AI in G5D/T CKD.

Since the research field is not too broad, we decided to include published conference proceedings. Two independent reviewers selected studies by screening the title and abstract. During the screening stage, 277 titles were excluded from the 354 papers previously deduplicated. Seventy-seven papers were found. In another phase, from the full articles which conformed to the selection criteria, essential data were extracted independently, and the results sorted. Discrepancies were resolved by discussion and consensus. Duplicates were excluded both manually and through reference manager software. Finally, 69 studies met the inclusion criteria (see Supplemental Tables 1–3).

### 2.2. Clinical Approach (according to the Clinical Topics)

**Figure 2:**
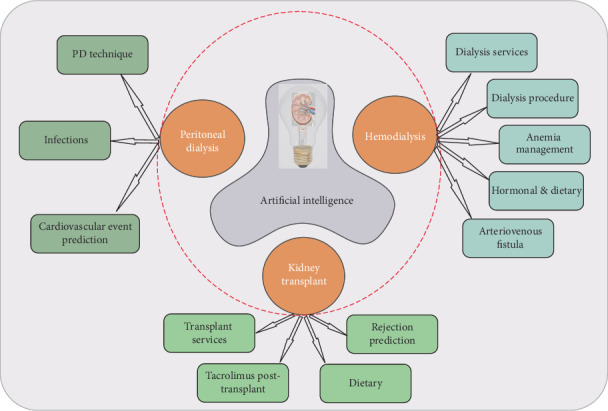
The involvement of AI in hemodialysis, peritoneal dialysis, and kidney transplant, respectively.

The sixty-nine included studies were split into three categories: AI and hemodialysis (HD), AI and peritoneal dialysis (PD), and AI and kidney transplantation (KT), respectively (Figure 2). There were 43 trials on AI in HD, eight studies in PD, and 18 in KT.

Moreover, each one of the three main categories was further divided into subsections (Table 1). Trials dealing with AI and HD covered five issues: (a) dialysis center/healthcare management, (b) dialysis technique and procedure, (c) anemia management, (d) hormonal/dietary issues, and (e) arteriovenous fistula assessment. Regarding PD, the studies were divided into three subsections: (a) peritoneal technique issues, (b) infections, and (c) cardiovascular event prediction. Finally, studies dealing with AI in KT were allocated into four categories: (a) healthcare management systems, (b) predicting graft rejection, (c) tacrolimus therapy modulation, and (d) dietary issues.

Most of the studies (except for one randomized controlled trial (RCT)) were observational. Only three trials were published before 2000, whereas over 60% of the studies were reported after 2010. Most HD studies involved personalized anemia management and parameters of the dialysis session. The accurate prediction of graft rejection or individualizing immunosuppressive treatment posttransplant was the main topics covered by the AI and KT trials.

### 2.3. Algorithm Approach (according to the AI/ML Algorithm Used)

All trials were also classified according to the type of AI algorithm (Table 2). Core concepts, various AI algorithms, and differences between them have been defined and described elsewhere [8–11].

Sixty-four studies included ML algorithms: unspecified, Naive Bayes models, support vector machine (SVM), and reinforcement learning with Markov decision processes (MDP). One study used *k*-nearest neighbor (*k*-NN), one study used multilayered perceptron (MLP), 30 studies used unspecified neural network algorithms, and 11 studies were based on tree-based modeling (TBM), random forest (RF), or conditional inference trees. Four trials used data mining algorithms, and five of them had fuzzy logic approaches. One study included specific natural language processing algorithms. Two studies have also included the Bayesian belief network and dynamic time warping (DTW) algorithms.

## 3. Discussions

Our endeavor is the first in-depth review of the literature gathering all studies using AI in dialysis or kidney transplantation. Two recent papers looked at AI in nephrology, but both focused on AI core concepts, AI perspectives, and algorithms [1, 8]. These articles explained in detail AI terminology and most of the algorithms used, also describing some significant clinical challenges, quoting only a few trials on dialysis and KT. Generally speaking, perspectives about AI oscillate between two extremes: either an extremely optimistic approach (“*algorithms are desperately needed to help*” [4]) or a disarming nihilism (“*Should we be scared of AI? Unfortunately, as our AI capabilities expand, we will also see it being used for dangerous or malicious purposes*” [12]).

### 3.1. Hemodialysis and AI

The most important implication of AI in HD is related to *dialysis services*. Generating auditing systems powered by AI/ML algorithms seems to improve major outcomes.

In a retrospective trial, 5800 dialysis sessions from 43 patients supervised for 19 months were assessed through temporal data mining techniques to gain insight into the causes of unsatisfactory clinical results [13]. Quality assessment was based on the automatic measurement of 13 variables reflecting significant aspects of a dialysis session, such as the efficiency of protein catabolism product removal or total body water (TBW) reduction and hypotension episodes. Based on the stratification of different causes of failed dialysis in time, ML algorithm “*learned*” association and temporal rules, reporting “*risk profiles*” for patients, containing typical failure scenarios [13].

Due to neural networks (NNs), we are now able to exclude the bias of the “*dialysis center effect*” on mortality [14] (the residual difference in mortality probability that exists between centers after adjustment for other risk factors). In a study including 18,000 ESRD patients from UK Renal Registry, an MLP (multilayered perceptron) was “*trained*” and then “*tested*” for predicting mortality. The authors proved with high accuracy that the renal center characteristics show little association with mortality and created a predictive survival model with a high degree of accuracy [14].

To have a real perception of what big data means, we present a study encompassing dialysis patients from the USRDS. A total of 1,126,495 records were included in a combined dataset, forty-two variables being selected to be used in the analysis based on their potential clinical significance. The authors described a feed-forward NN with two inputs, one output, and a hidden layer containing four neurons. A powerful tool was created to predict mortality with high accuracy [15].

In daily practice, clinician nephrologists could also use forecast models that can predict the quality of life (QoL) changes (through an early warning system performing dialysis data interpretation using classification tree and Naive Bayes) [16] and cardiovascular outcomes (a lasso logistic regression model and an RF model were developed and used for predictive comparison of data from 4246 incident HD patients) [17].

Probably the most attractive and tempting field is the *HD session*. An actual dialysis machine cannot adapt/react when various changes occur; only AI may lead to personalized “*precision medicine*” [18]. Indeed, a recent AI model was able to predict hypotension and anticipate patient's reactions (in terms of volumes, blood pressure, and heart rate variability) [7]. Since the NN approach is more flexible and adapts easier to complex prediction problems compared to standard regression models, the authors included 60 variables (patient characteristics, a historical record of physiological reactions, outcomes of previous dialysis sessions, predialysis data, and the prescribed dialysis dose for the index session). The dataset used for modeling consisted of 766,000 records, each representing a dialysis session recorded in the Spanish NephroCare centers. NN proved to be a better predictor than a standard recommendation from the guidelines regarding the urea removal ratio, postdialysis BUN, or Kt/V [19]. ML algorithms were used to predict low blood pressure [20], blood volume [21, 22], or TBW [23].

Recent studies suggest that NN outperforms experienced nephrologists. A combined retrospective and prospective observational study was performed in two Swiss dialysis units (80 HD patients, 480 monthly clinical and biochemical variables). A NN was “*trained*” and “*tested*” using the BrainMaker Professional software, predicting intradialytic hypotension better than six trained nephrologists [20]. In other studies, 14 pediatric patients were switched from nephrologists to AI. Results proved that AI is a superior tool for predicting dry weight in HD based on bioimpedance, blood volume monitoring, and blood pressure values [24, 25].

Since *anemia* is a frequent comorbidity found in ESRD and dialysis patients [26], key elements were targeted by AI software [26]: erythropoietin-stimulating agents [27], hemoglobin target [28], and iron treatment dosing [29].

An impressive recent retrospective observational study included HD patients from Portugal, Spain, and Italy from 2006 to 2010 in Fresenius Medical Care clinics. At every treatment, darbepoetin alpha and iron dose administration was recorded as well as parameters concerning HD treatment. These data represent the input for “*training*” and “*testing*” a feed-forward multilayered perceptron (MLP). This approach puts together the potential of MLP to produce accurate models given a representative dataset with better use of the available information using a priori knowledge of RBC lifespan and the effect produced by iron and ESA [28]. Other studies also used NNs to individualize ESA dosage in HD patients with outstanding results [30, 31]. Moreover, NN implemented in clinical wards could appreciate erythropoietin responsiveness [32–34].

Probably the most advanced system to manage anemia in HD is the “*Anemia Control Model*” (ACM) [35]. Conceived and validated previously [28], this model can predict future hemoglobin and recommend a precise ESA dosage. It was deployed in 3 pilot clinics as part of routine daily care of a large population of unselected patients. Also, a direct comparison between standard anemia management by expert nephrologists following established best clinical practices and ACM-supported anemia management was performed. Six hundred fifty-three patients were included in the control phase and 640 in the observation phase. Compared to the “*traditional*” management, the AI approach led to a significant decrease in hemoglobin fluctuation and reductions in ESA use, with the potential to reduce the cost of treatment [35].

AI algorithms also improve chronic kidney disease-mineral and bone disorder (CKD-MBD) management in HD. Some studies suggest that NN (based on limited clinical data) can accurately forecast the target range of plasma iPTH concentration [36]. An MLP was constructed with six variables (age, diabetes, hypertension, hemoglobin, albumin, and calcium) collected retrospectively from an internal validation group (*n* = 129). Plasma iPTH was the dichotomous outcome variable, either target group (150 ng/L ≤ iPTH ≤ 300 ng/L) or nontarget group (iPTH < 150 ng/L or iPTH > 300 ng/L). After internal validation, the ANN was prospectively tested in an external validation group (*n* = 32). This algorithm provided excellent discrimination (AUROC = 0.83, *p* = 0.003) [36]. Usually, frequent measurement is needed to avoid inadequate prescription of phosphate binders and vitamin D. AI can repeatedly perform the forecasting tasks and may be a satisfactory substitute for laboratory tests [37]. NNs were used to predict HD patients who need more frequent vitamin D dosage, using only simple clinical parameters [38]. However, analysis of the complex interactions between mineral metabolism parameters in ESRD may demand a more advanced data analysis system such as random forest (RF) [39].

Another work based on natural language processing algorithms applied to 11,451 USRDS patients described the real incidence and mortality of calciphylaxis patients [40]. It identified 649 incident calciphylaxis cases over the study period, with mortality rates noted to be 2.5–3 times higher than average mortality rates. This trial serves as a template for investigating other rare diseases. However, the algorithm in this paper has not been disclosed by the authors.

*Patency of the arteriovenous access* is the last aspect involving AI in HD. Fuzzy Petri net (FPN) algorithms were involved in quantifying the degree of AV fistula stenosis. A small study from Taiwan (42 patients) used an electronic stethoscope to estimate the characteristic frequency spectra at the level of AV fistula. Observing three main characteristic frequencies, it provides information to evaluate the degree of AVS stenosis [41].

Moreover, the life of the AV fistula could be forecasted by ML algorithms [42]. Six ML algorithms were compared to predict the patency of a fistula based on clinical and dialysis variables only. Of these, SVM with a linear kernel gives the highest accuracy of 98%. The proposed system was envisioned after considering the dataset of 200 patients, five dialysis sessions for each patient (to avoid any operator error), and over 30 values reported by the machine. Such a system may improve the patient's QoL by foregoing the catheter and reducing the medical costs of scans like ultrasound Doppler [42].

Finally, another study using small-sized sensors (as opposed to conventional Doppler machines) used SVM for assessing the health of arteriovenous fistula. The model achieved high accuracy (89.11%) and a low type II error (9.59%) [43].

#### 3.1.1. Key Messages

*(1) How Can the Use of AI Improve Healthcare Delivery to HD?*.

(i) Prevention
  - AI methods capable of determining risk profiles for unsatisfactory clinical results of HD sessions were described
  - Early detection allows for timely correction of risk factors to attain good quality HD sessions and favorable outcomes
(ii) Diagnosis
  - Estimating the patency of AV fistula by AI approaches may improve HD session outcomes and the patient's QoL
  - AI solutions reduce medical costs by replacing more expensive diagnostic procedures
(iii) Prescription
  - AI can recommend medication dosage for preventing HD-specific complications like anemia and hemoglobin fluctuations, mineral imbalance
  - Algorithm involvement leads to fewer complications and reduces medication use, proving the potential to reduce treatment expenses
(iv) Prediction
  - AI was used for predicting mortality and survival in HD
  - Specific algorithms predict changes in QoL, cardiovascular outcomes, and intradialytic hemodynamic events
  - Survival and QoL predictive models can help mitigate the impact on public health by better directing the use of resources
  - Predicting intradialytic events allows for flexible adaptions of the HD process in real time by avoiding hypotension, the variability of heart rate and volumes, thus ensuring the success of the HD session and overall cost efficiency of interventions

*(2) Challenges and Areas Which Require More Studies in AI for HD*.

- Real-time monitoring AI systems could achieve personalized treatment with embedded automatic adaptive responses in HD sessions
- Implementing potential interaction through feedback between AI/ML systems and physicians responsible for HD would allow both parts to learn from each other and provide better decisions for ESRD patients
- The more AI systems will be deployed in HD patient care, the larger the scale data will be available. This should compel to the development of stringent regulations concerning data privacy, maintenance, and sharing for safer implementation in public healthcare

### 3.2. Peritoneal Dialysis and AI

One of the first applications of AI in PD was the selection of *PD schemes*. Fuzzy logic algorithms were used in small studies with excellent compatibility with doctors' opinions [44]. Since high peritoneal membrane transport status is associated with higher morbidity and mortality, determining peritoneal membrane transport status can result in a better prognosis. An MLP used predialysis data from a 5-year PD database of 111 uremic patients and demonstrated the usefulness of this approach to stratify predialysis patients into high and low transporter groups [45]. The evaluation of peritoneal membrane transport status, if predictable before PD, will help clinicians offer their uremic patients better therapeutic options.

Almost 40% of PD patients experience technique failure in the 1^st^ year of therapy. Understanding which factors are genuinely associated with this outcome is essential to develop interventions that might mitigate it. Such data were obtained from a high-quality registry—UK Renal Registry [46]: between 1999 and 2004, 3269 patients were included in the analysis. An MLP with 73-80-1 nodal architectures was constructed and trained using the back-propagation approach.

Due to the vast number of data acquired from continuous ambulatory peritoneal dialysis (CAPD) patients (routine lab tests on follow-up), data mining algorithms were proposed to discover patterns from meaningless data (e.g., consecutive creatinine values) [47]. This study is probably one of the best examples of AI in medicine: identifying patterns in big data series.

AI/ML algorithms would help predict impending complications such as fluid overload, heart failure, or peritonitis, allowing early detection and interventions (remote patient management) to avoid hospitalizations [48].

Using a systematic approach to characterize responses to microbiologically well-defined *infection* in acute peritonitis patients, ML techniques were able to generate specific biomarker signatures associated with Gram-negative and Gram-positive organisms and with culture-negative episodes of unclear etiology [49]. By combining biomarker measurements during acute peritonitis and feature selection approaches based on SVM, NN, and RF, a study (including 83 PD patients with peritonitis) demonstrated the power of advanced mathematical models to analyze complex biomedical datasets and highlight critical pathways involved in pathogen-specific inflammatory responses at the site of infection.

Using data mining models in CAPD patients, patterns were extracted to classify a patient with stroke risk, according to their blood analysis [50]. In a recent study analyzing a dataset from 850 cases, five different AI algorithms (Naïve Bayes, Logistics Regression, MLP, Random Tree, and *k*-NN) were used to predict the stroke risk of a patient. The specificity and sensibility of RT and *k*-NN were 95% in predicting stroke risk. Shortly, PD patients will benefit from a high prediction (stroke, infection, cardiovascular events [51], or even mortality risk) only from information easy to obtain (demographical, biological, or PD-related data).

#### 3.2.1. Key Messages

*(1) How Can the Use of AI Improve Healthcare Delivery to PD?*.

(i) Prevention
  - AI was used to identify factors associated with PD technique failure
  - Developing interventions to mitigate risk factors to prevent this outcome can be the result of the critical contribution of AI in the PD process
(ii) Diagnosis
  - AI algorithms found specific biomarker signatures associated with different types of infections
  - This has significant implications in the early initiation of appropriate treatment and in avoiding severe infectious complications of the vulnerable ESRD patients
  - AI contributed to expanding scientific knowledge of the pathophysiological mechanisms by highlighting critical pathways involved in pathogen-specific inflammatory responses in PD
(iii) Prescription
  - By using AI, better therapeutic options can be offered to uremic patients by stratifying predialysis patients into high and low transporter groups
  - This will improve prognosis and reduce morbidity and mortality in PD patients
(iv) Prediction
  - AI can predict complications such as fluid overload, heart failure, or peritonitis
  - Also, algorithms could identify patients with stroke risk, thereby allowing early interventions and reduce PD hospitalizations

*(2) Challenges and Areas Which Require More Studies in AI for PD*.

- AI could be exploited to identify patients at risk of developing peritonitis, a significant complication of PD, in order to reduce the infectious risk and to overcome a substantial burden in the PD process
- Conducting studies on home remote monitoring in automated PD may improve patients' outcomes and adherence to the therapy
- While dialysate regeneration using sorbent technology makes it possible to build an automated wearable artificial kidney PD device, studies of safety in this area would be much needed, especially for patients facing mobility problems

### 3.3. Kidney Transplantation and AI

Given the high number of dialysis patients and the limited number of organ donors, AI algorithms are involved in optimizing *the healthcare management system* [52]. Through data mining and NN algorithms, complex e-health systems are proposed for a wiser allocation of organs and predicting transplant outcomes [53].

The most stirring contemporary issue regarding KT is the power of AI to *predict graft rejection*. A study published 20 years ago reported that NNs could be utilized in the prediction of chronic renal allograft rejection (the authors described a retrospective analysis on 27 patients with chronic rejection, eight simple variables manifesting a strong influence on rejection) [54]. Another cohort study of 500 patients from 2005 to 2011 used ML algorithms (SVM, RF, and DT) to predict “*delayed graft function*.” Linear SVM had the highest discriminative capacity (AUROC of 84.3%), outperforming the other methods [55]. However, currently, no guideline supports the use of AI in organ allocation or prediction of rejection.

Despite ongoing efforts to develop other methods, serum creatinine remains the most important parameter for assessing renal graft function. A rise in creatinine corresponds to deterioration in the KT function. The physician should recognize “*significant*” increases in serum creatinine. DTW was used to identify abnormal patterns in a series of laboratory data, thus detecting earlier and reporting creatinine courses associated with acute rejection [56]. Data extraction was performed on 1,059,403 laboratory values, 43,638 creatinine measurements, 1143 patients, and 680 rejection episodes stored in the database. By integrating AI into the electronic patient registration system, the real impact on the care of transplant recipients could be evaluated prospectively.

An MLP trained with back-propagation was used in a retrospective study on 257 pediatric patients who received KT to identify delayed decrease of serum creatinine (a delay in functional recovery of the transplanted kidney) using 20 simple input variables [57]. Other models (decision trees) were reported to highlight subjects at risk of graft loss [58].

Using datasets from the USRDS database (48 clinical variables from 5144 patients), other authors developed an ML software (based on Bayesian belief network (BBN)) that functioned as a pretransplant organ-matching tool. This model could predict graft failure within the first year with a specificity of 80% [59]. Other stand-alone software solutions (trained on big data) incorporated pretransplant variables and predicted graft loss and mortality [60–62]. Since these AI tools can be easily integrated into electronic health records, we appreciate that all kidney transplants will be managed with AI tools in the next years.

Few studies were found dealing (through AI algorithms) with *posttransplant immunosuppressive therapy*.

The objective of a study was to identify adaptation rules for tacrolimus therapy from a clinical dataset to predict drug concentration [63]. Since tacrolimus has a narrow therapeutic window and variability in clinical use, the challenge for various ML models was to predict the tacrolimus stable dose (TSD). In a large Chinese cohort comprising 1045 KT patients, eight ML techniques were trained in the pharmacogenetic algorithm-based prediction of TSD. Clinical and genetic factors significantly associated with TSD were identified. Hypertension, use of omeprazole, and CYP3A5 genotype were used to construct the multiple linear regression (MLR) [55].

A prospective study involving 129 KT patients confirms that the combination of multiple ABCB1 polymorphisms with CYP3A5 genotype through a NN calculates more precisely the initial tacrolimus dose improving therapy and preventing tacrolimus toxicity [64].

Finally, the only RCT reported in our review was a study evaluating the benefits of different *types of diets after transplant* [65]. NN seems to be the most suitable method for investigations with many variables, interconnected nonlinearly, allowing for a more general approach to biological problems. 37 KT patients were randomized either to a low-fat standard or a Mediterranean diet (MD). For the MD group, the NNs had two hidden layers with 223 and 2 neurons. In the control group, the networks had two hidden layers as well, with 148 and 2 neurons, respectively. The conclusion was that MD would be ideal for posttransplant patients, without affecting the lipid profile.

#### 3.3.1. Key Messages

*(1) How Can the Use of AI Improve Healthcare Delivery to KT?*.

(i) Diagnosis
  - AI was able to detect and report early creatinine courses associated with acute KT rejection by identifying abnormal patterns in a series of laboratory data, thus allowing for rapid intervention and improved aftermath in KT patients
(ii) Prescription
  - Various ML models accurately predict the tacrolimus stable dose succeeding to improve posttransplant immunosuppressive therapy and prevent tacrolimus toxicity
  - Proper management of immunosuppression can have a significant impact on averting graft loss
  - ML can evaluate the benefits of different types of diets after transplant that can lead to a positive impact on QoL in KT
(iii) Prediction
  - AI is used to predict graft rejection, “delayed graft function,” and mortality
  - AI algorithm—pretransplant organ-matching tool
  - This allows for the wiser allocation of organs and overall optimization of the healthcare management system in KT

*(2) Challenges and Areas Which Require More Studies in AI for KT*.

- Preventive AI tools could certainly be employed in identifying modifiable risk factors for graft rejection and graft loss, offering patients better chances for successful KT
- Guidelines need to be developed for supporting the use of AI in organ allocation or prediction of rejection
- Prospective evaluation of the real AI impact on the care of transplant recipients can be easily accomplished by integrating AI into electronic patient registration systems
- We appreciate that in the next years, all kidney transplant procedures will be managed through AI tools

### 3.4. Internet of Things (IoT) and Wearables in Dialysis/Transplantation Management

The emergence of the modern IoT concept yielded the idea of connecting everything to the Internet and generating data through sensors' signals regarding external information and changes in the environment, thus moving AI to the edge in healthcare.

IoT wearable systems are capable of real-time remote monitoring and analyzing HD and PD patients' physiological parameters by integrating sensors to detect a pulse, temperature [66], blood pressure [67], blood leakage [68, 69], electrocardiographic measurements, hyperkalemia, or fluid overload [70].

Medical wearables have been proposed to help and support KT patients in aftercare by monitoring vital signs, heart rate, temperature, blood pressure, physical activity, and risk calculations [71].

IoT medical devices have a tremendous impact on healthcare monitoring and treatment outcomes. They collect a substantial amount of data that can be employed in training new ML models for providing better patient care. Nevertheless, large-scale data can make them vulnerable to security breaches; thus, privacy and security issues need to be rigorously addressed.

## 4. Limitations and Future Directions

Probably the most important limitation of the AI/ML approach is that there is a need for robust validation in real-world studies. We are aware that AI enthusiasm exceeds AI software abilities, mainly due to a lack of clinical validation and daily care implementation.

Since ML algorithms have the strengths to learn and improve from experience without being explicitly programmed for a specific task, AI is often perceived as a “*black box*” that hides the precise way in which it concludes/results [72]. Due to the “*hidden layers*” of a NN, likely, the algorithm itself cannot adequately describe the decision-making process, none being like the previous one. Currently, there is a real debate whether it is acceptable to use nontransparent algorithms in a clinical setting (the so-called “*deconvolution*” of algorithms being required by the European Union's General Data Protection Regulation) [4].

There are more questions about how an ML algorithm discovers patterns and learns from a dataset on predicting dialysis volumes than on how AI is used in self-driving cars. In a recent editorial, we are underlining a novel paradigm used to optimize the diagnostic and treatment of ESRD, inviting clinicians and researchers to envision and expect more from AI.

However, the reluctance of the medical community in implementing AI in clinical practice derives from the reliance on RCTs—the cornerstone of Evidence-Based Medicine (EBM). It seems that the transition from the “*EBM paradigm*” to the “*deep medicine*” concept is confusing, burdensome, and meets new and unexpected obstacles. Another disadvantage of NNs is that inputs and outputs of the network are often surrogates for clinical/paraclinical situations. Surrogates should be particularly relevant to maximizing the correlation between the prediction of the network and the clinical situation. This correlation may not always be optimal [8].

The privacy of personal information and the security of data are an important matter. It is difficult to provide security guarantees regarding the risk of hacking and manipulating content, events that could limit the progress of AI in medicine. More than that, due to private corporations' cooperation and investment in AI medical projects, there is a serious risk (and fear) that doctors will be constrained to work on AI machines that they do not understand or trust, manipulating the medical data subsequently stored on private servers. This has led one of the largest private-sector investors to claim that AI is far more dangerous than nukes.

Finally, when programmers approach a problem related to HD or transplantation, there is still debate over the superiority of one algorithm over another. The tendency is to demonstrate the superiority of deep learning algorithms through ML, but the soundness of this approach requires long periods of “*training*” and large databases.

There is a chance that the entire dialysis process will be monitored and predicted by AI solutions. Future dialysis complications will be foreseen through simple clinical/paraclinical variables. Mining knowledge from big data registries will allow building intelligent systems (the so-called Clinical Decision Support Systems), which will help physicians in classifying risks, diagnosing CKD 5D/T complications, and assessing prognosis [73].

## 5. Conclusions

Although the guidelines are reluctant to recommend the implementation of AI in daily clinical practice, there is evidence that AI/ML algorithms can predict better than nephrologists: volumes, Kt/V, and risk of hypotension and cardiovascular events during dialysis. There are integrated anemia management AI systems, through personalized dosing of ESA, iron, and hemoglobin modulation. Recent studies employ ML algorithms as pretransplant organ-matching tools, thus minimizing graft failure and accurately predicting mortality. Altogether, these trials report a significant impact of AI on quality of life and survival in G5D/T patients. In the coming years, we will probably witness the emergence of AI/ML devices that will improve the management of dialysis patients.

## Figures and Tables

**Table 1:** AI studies involved in HD, PD, and KT, respectively.

Stage G5 CKD	Clinical issue	No.	Studies	No.	Ref.
HD					
a	Dialysis services	7	Bellazzi 2005, Raghavan 2005, Tangri 2006, Jacob 2010, Titapiccolo 2013, Saadat 2017, Usvyat 2018	43	[13–17, 74, 75]
b	Dialysis procedure	14	Nordio 1994, Nordio 1995, Guh 1998, Akl 2001, Goldfarb-Rumyantzev 2003, Gabutti 2004, Chiu 2005, Fernandez 2005, Mancini 2007, Cadena 2010, Azar 2011, Niel 2018, Barbieri 2019, Hueso 2019		[20–23, 76–78] [7, 18, 19, 25, 79–81]
c	Anemia management	13	Martin Guerrero 2003, Martin Guerrero 2003, Gabutti 2006, Gaweda 2008, Gaweda 2008, Fuertinger 2013, Escandel-Montero 2014, Barbieri 2015, Barbieri 2016, Barbieri 2016, Brier 2016, Brier 2018, Bucalo 2018		[26–28, 30–34] [29, 35, 82, 83]
d	Hormonal & dietary	6	Gabutti 2004, Wang 2006, Chen 2007, Bhan 2010, Nigwekar 2014, Rodriguez 2016		[20, 36–40]
e	AV fistula	3	Chen 2014, Bhatia 2018, Chao 2018		[41–43]
PD					
a	Peritoneal technique	5	Zhang 2005, Chen 2006, Tangri 2011, Brito 2019, John 2019	8	[44–48]
b	Infections	1	Zhang 2017		[49]
c	Cardiovascular events	2	Rodriguez 2017, Fernandez- Lozano 2018		[50, 51]
KT					
a	Healthcare management	2	Sharma 2008, Karademirci 2015	18	[52, 53]
b	Rejection prediction	11	Simic-Ogrizovic 1999, Fritsche 2002, Santori 2007, Greco 2010, Brown 2012, Decruyenaere 2015, Srinivas 2017, Yoo 2017, Gallon 2018, Jia 2018, Rashidi Khazaee 2018		[54, 56–62, 84–86]
c	Tacrolimus post-T	4	Seeling 2012, Tang 2017, Niel 2018, Thishya 2018		[55, 63, 64, 87]
d	Dietary	1	Stachowska 2006		[65]

**Table 2:** Different types of AI algorithms used in G5D/T trials.

Type of AI/ML algorithm used	No.	Studies	Ref.
Unspecified machine learning (ML) algorithms	15	Cadena 2010, Fuertinger 2013, Barbieri 2015, Barbieri 2016, Brier 2016, Saadat 2017, Bhatia 2018, Bucalo 2018, Usvyat 2018, Zhang 2017, John 2019, Decruyenaere 2015, Karademirci 2015, Tang 2017, Gallon 2018	[27, 28, 35, 80] [16, 42, 82, 83] [48, 49, 75, 84] [53, 55, 85]
ML—Naive Bayes models	1	Rodrigues 2017	[50]
ML—support vector machine (SVM)	3	Martin-Guerrero 2003, Chao 2018, Fernandez-Lozano 2018	[30, 43, 51]
ML—*k*-NN (*k*-nearest neighbor)	1	Fernandez-Lozano 2018	[51]
ML—reinforcement learning with Markov decision processes (MDP)	1	Escandell-Montero 2014	[26]
Fuzzy			
Fuzzy logic	5	Nordio 1994, Nordio 1995, Mancini 2007, Gaweda 2008, Zhang 2005	[21, 22, 34, 79] [44]
Coactive fuzzy	1	Chen 2007	[37]
Fuzzy Petri nets	1	Chen 2014	[41]
ML—natural language processing	1	Nigwekar 2014	[40]
Data mining	4	Bellazzi 2005, Brito 2019, Srinivas 2017, Jia 2018	[13, 47, 60, 86]
Bayesian belief network	1	Brown 2012	[59]
Dynamic time warping (DTW)	1	Fritsche 2002	[56]
ML—unspecified neural network (NN) algorithm	30	Guh 1998, Akl 2001, Goldfarb-Rumyantzev 2003, Martin Guerrero 2003, Gabutti 2004, Gabutti 2004, Chiu 2005, Fernandez 2005, Gabutti 2006, Tangri 2006, Wang 2006, Gaweda 2008, Bhan 2010, Jacob 2010, Azar 2011, Barbieri 2016, Brier 2018, Niel 2018, Barbieri 2019, Hueso 2019, Chen 2006, Tangri 2011, Simic-Ogrizovic 1999	[31, 76–78] [19, 20, 23, 88] [14, 32, 33, 36] [15, 29, 38, 81] [7, 18, 87, 89] [45, 46, 54, 65]
ML—multilayer perceptron		Stachowska 2006, Santori 2007, Sharma 2008, Tang 2017	[25, 52, 55, 57]
(MLP) ML—recurrent NN		Niel 2018, Rashidi Khazaee 2018, Thishya 2018	[62, 64]
	1	Martin-Guerrero 2003	[31]
	1	Gallon 2018	[85]
ML—tree-based modeling (TBM)			
Random forest (RF)	7	Titapiccolo 2013, Rodriguez 2016, Fernandez-Lozano 2018, Sharma 2008, Greco 2010, Tang 2017, Yoo 2017	[17, 39, 51, 52] [55, 58, 61]
Decision trees	3	Goldfarb-Rumyantzev 2003, Raghavan 2005, Yoo 2017	[61, 74, 78]
Conditional inference trees	1	Seeling 2012	[63]

## Supplementary Materials

Supplementary Materials Supplemental Table 1: short description and results of the studies involving AI in HD. Supplemental Table 2: short description and results of the studies involving AI in PD. Supplemental Table 3: short description and results of the studies involving AI in KT
