# Supplementary material for: Using Artificial Intelligence Resources in Dialysis and Kidney Transplant Patients: A Literature Review
Source: Biomed Res Int. 2020 Jun 10;2020:9867872. doi: 10.1155/2020/9867872 (PMC7303737; doi:10.1155/2020/9867872)
Supplement: Supplementary Materials — Supplemental Table 1: short description and results of the studies involving AI in HD. Supplemental Table 2: short description and results of the studies involving AI in PD. Supplemental Table 3: short description and results of the studies involving AI in KT [file 9867872.f1.pdf]

Supplemental Table 1.

Author	Year	Title of study	Type of AI/ML algorithm used	Type of study & No HD patients	Results
Nordio, M.	1994	Projection and simulation results of an adaptive fuzzy control module for blood pressure and blood volume during HD	Integrative discrete-time fuzzy control module for blood pressure and blood volume	Observational study<br><br>10 HD patients	The adaptive control system was simulated on an IBM PC, and rules and terms were expressed by linguistic judgments such as: IF "situation", THEN "action". A pre-processor converted the rules into the numerical values rendered in the tables. The simulation results thus obtained were satisfactory, while the introduction of Na control allowed the achievement of the target dry weight of the patient with a stable blood pressure.
Nordio, M.	1995	A new approach to blood pressure and blood volume modulation during HD: an adaptive fuzzy control module	Discrete-time fuzzy control module	Observational study<br><br>10 HD patients	A smooth function of volemia acted on the second control variable, Na concentration in the dialysate. The adaptive control system was simulated on an IBM-PC, rules and terms were expressed by linguistic judgments such as: IF "situation", THEN "action". A pre-processor converted the rules into the numerical values rendered in the reference tables. The obtained simulation results were satisfactory, the introduction of the Na control allowed reaching the target dry weight of the patient with a stable blood pressure.
Guh, J. Y.	1998	Prediction of equilibrated postdialysis BUN by an ANN in high-efficiency HD	ANN was used to predict the equilibrated BUN (Ceq) and equilibrated Kt/V (eKt/V60) by using both pre-dialysis, post-dialysis and low-flow post-dialysis BUN.	Observational study<br><br>74 patients on high-efficiency or high-flux HD	In patients with a high urea rebound (>30%), although Smye formula lost its accuracy, low-flow ANN remained accurate.<br><br>In the prediction of eKt/V60, both Daugirdas' formula and low-flow ANN were equally accurate, although the Smye formula was less so.<br><br>In patients with a high urea rebound, although both Smye and Daugirdas' formulas lost their accuracy, low-flow ANN remained accurate.<br><br>Low-flow ANN can accurately predict both Ceq and eKt/V60 regardless of the degree of urea rebound.
Akl, A. I.	2001	AI: a new approach for prescription and monitoring of HD therapy	AI using neural networks (NNs) studied and predicted concentrations of urea during a HD session	Observational study<br><br>15 chronic HD patients	Comparing results of the NN model with the DDQ (direct dialysate quantification) model, the prediction error was 10.9%, with a non-significant difference between predicted total urea nitrogen (UN) removal and measured UN removal by DDQ. NN model predictions of time showed a non-significant difference with actual intervals

					needed to reach the same SRI (solute removal index) level at the same patient conditions, except for the prediction of SRI at the first 30-minute interval, which showed a significant difference (p= 0.001).
Goldfarb-Rumyantzev, A.	2003	Prediction of single-pool Kt/v based on clinical and HD variables using multilinear regression, tree-based modeling, and ANNs	Multilinear regression (LM), tree-based modeling (TBM), and ANNs to predict actual spKt/V.	Observational study<br><br>602 HD patients	Prediction algorithms were developed from a "training" dataset and were validated on a separate "testing" dataset.<br><br>Correlation coefficients of predicted spKt/V with measured spKt/V with and without nPNA (total nitrogen appearance) respectively were 0.745 and 0.679 for LM, 0.6 and 0.512 for TBM, and 0.634 for ANN, which performed better without using nPNA.
Martin Guerrero, J. D.	2003	Use of ANNs for dosage individualisation of erythropoietin in patients with secondary anemia to chronic renal failure	Neural models for dosage individualisation of erythropoietin in CKD patients with secondary anemia	Observational study<br><br>110 HD patients	Neural models carried out an individualised prediction of the erythropoietin dosage to be administered to patients with secondary anemia due to chronic renal failure undergoing periodic HD<br><br>Since the results obtained were excellent, an easy-to-use decision-aid computer application was implemented.
Martin-Guerrero, J. D.	2003	Dosage individualization of erythropoietin using a profile-dependent support vector regression	The support vector regressor (SVR) was benchmarked with the classical multilayer perceptron (MLP) and the Autoregressive Conditional Heteroskedasticity (ARCH) model for an individualized prediction of the EPO dosage to be administered to chronic renal failure in periodic HD	Observational study<br><br>110 HD patients	The so-called profile-dependent SVR (PD-SVR) displayed improved results of the standard SVR method and the MLP.<br><br>The sensitivity analysis on the MLP and inspection the distribution of the support vectors in the input and feature spaces were useful for gaining knowledge about the issue.
Gabutti, L.	2004	Usefulness of ANNs to predict follow-up dietary protein intake in HD patients	ANNs were useful tools in HD to predict the follow-up (1 month after the observation used for the prediction) of dietary protein intake (PCR)	Retrospective and prospective observational study<br><br>84 HD patients	ANN compared with nephrologists gave a more accurate correlation between estimated and calculated follow-up PCR (P < 0.001).<br><br>ANN was seen in the ability to detect a follow-up PCR <1.00 g/kg/day expressed as a percentage of correct predictions, sensitivity, specificity, and predictability.

					The inter-institutional performance of the ANN was positively influenced by the size and the variability of the population used to build the mathematical model. The size of the population selected to build the ANN was critical for its performance.
Gabutti, L.	2004	ANNs improve the prediction of Kt/V, follow-up dietary protein intake and hypotension risk in haemodialysis patients	ANNs were useful to predict the dialysis quality (Kt/V), the follow-up dietary protein intake and the risk of intradialytic hypotension in HD patients	Combined retrospective and prospective observational study<br><br>80 HD patients	ANN compared with nephrologists gave a more accurate correlation between estimated and calculated Kt/V and follow-up PCR ($p<0.001$).<br><br>ANN was seen in the ability to detect a Kt/V < 1.30, a follow-up PCR < 1.00 g/kg/day and the occurrence of hypotension expressed as a percentage of correct answers, sensitivity, specificity and predictability.
Bellazzi, R.	2005	Temporal Data Mining for the quality assessment of HD services	Intelligent data analysis and temporal data mining techniques were applied to gain insight and knowledge on the causes of unsatisfactory clinical results	Observational retrospective study<br><br>5800 dialysis sessions of 43 different patients monitored for 19 months	The new methods proposed were suitable tools for knowledge discovery in clinical time series. Their use in the context of an auditing system for dialysis management helped clinicians improve their understanding of the patients' behavior.
Chiu JS	2005	Applying an ANN to predict TBW in HD	The predictive value of TBW (total body water) based on ANN and five anthropometric equations - 58% of actual body weight, Watson formula, Hume formula, Chertow formula, and Lee formula	Observational study<br><br>54 HD patients	ANN could surpass traditional anthropometric equations and served as a feasible alternative method of TBW estimation for chronic HD patients.
Fernandez, E. A.	2005	Comparison of different methods for HD evaluation by means of ROC curves: from AI to current methods	AI Method based on an ANN	Observational study<br><br>113 HD patients	For both cases (URR and Kt/V), the minimum doses required to achieve the lowest FPR (false positive rate) and ER (error rate) for the standard methods (stdURR and Kt/Vsp) were higher than those reported by the DOQI guidelines, being 70% for stdURR and 1.35 for Kt/Vsp, whereas for those methods using the double-pool Kt/V or equilibrated URR, the dose targets were close to those recommended by DOQI and ERA.

					The method for target dose selection was easy to understand, and it took into account both accuracy and confidence of the adequacy tool.<br><br>ANN method was identified to be superior to the Smye method for estimation of equilibrated urea, the results suggesting that ANN methods could be useful tools in the analysis of nephrology data.
Raghavan, S. R.	2005	Developing decision support for dialysis treatment of chronic kidney failure	An intelligent system building on existing knowledge and susceptible to reconfiguration on the basis of knowledge acquired during the use of the DARWIN information system	Observational study<br><br>634 HD patients	The problems faced by practitioners in managing chronic kidney failure patients contributed to the decision for support techniques used in developing a dialysis decision support system.<br><br>The DARWIN decision support design process demonstrated the feasibility of implementing flexible clinical decision support using standard software tools.<br><br>The DARWIN decision support system was developed using Microsoft Active Server Pages, Visual Basic, and Microsoft SQL Server as the database.
Gabutti, L.	2006	Would ANNs implemented in clinical wards help nephrologists in predicting epoetin responsiveness?	Performance of ANNs in predicting the dose required to reach the hemoglobin target and for the monthly dose adjustments	Prospective, non-randomized observational study<br><br>340 HD patients of 26 centers	For a specificity of 50%, the sensitivity of ANNs compared to linear regressions in predicting the erythropoietin dose to reach the hemoglobin target was 78 vs. 44% ($p < 0.001$).<br><br>The ANN built to predict the monthly adaptations in erythropoietin dose, compared with the nephrologists' opinions, allowed to detect 48 vs. 25% ($p < 0.05$) of the patients treated with an insufficient dose with a specificity of 92 vs. 83% ($p < 0.05$).<br><br>In predicting the erythropoietin dose required for an individual patient and the monthly dose adjustments, ANNs were superior to the nephrologists' opinions. ANN may be a useful and promising tool that could be implemented in clinical wards to help nephrologists in prescribing erythropoietin.
Tangri, N.	2006	Lack of a centre effect in UK renal units: application of an ANN model	An ANN model to predict mortality within 1 year in UK Renal Registry (UKRR)	Cohort study<br><br>18 015 prevalent ESRD patients	A highly efficient model for predicting the mortality within 1 year was created after restricting the model to use demographic and case-enriched data [area under the receiver operating characteristic curve (AUROC) = 0.974].<br><br>The addition of the dialysis centre code and centre size as input variables did not add to the efficiency of the model (AUROC = 0.962).

					Dialysis centre code or size alone was not predictive of mortality when applied to an artificial neuronal network architecture (AUROC = 0.649 and 0.628).
Wang, Y. F.	2006	Prediction of target range of intact parathyroid hormone in HD patients with ANN	ANN was a useful tool for predicting the target range of plasma intact parathyroid hormone (iPTH) concentration	Observational study<br><br>Internal validation group: 129 HD patients<br><br>External validation group: 32 HD	The externally validated ANN provided excellent discrimination as appraised by area under the receiver operating characteristic curve (0.83 ± 0.11, p = 0.003).<br><br>ANN, which is based on limited clinical data, was able to accurately forecast the target range of plasma iPTH concentration in HD patients.
Chen, C. A.	2007	Neuro-fuzzy technology as a predictor of parathyroid hormone level in HD patients	A coactive neuro-fuzzy inference system (CANFIS) to predict plasma PTH concentrations	Cohort study<br><br>121 HD patients	Plasma PTH concentrations measured by RIA (radioimmunoassay) and predicted by CANFIS were 179.04 +/- 38.18 ng/l and 179.34 +/- 37.76 ng/l, respectively (p = 0.15).<br><br>The CANFIS was able to precisely estimate plasma PTH concentrations in HD patients.<br><br>The neuro-fuzzy technology, based on limited clinical parameters, was an excellent alternative to RIA for accurately predicting plasma PTH concentration in HD patients.
Mancini, E.	2007	Prevention of dialysis hypotension episodes using fuzzy logic control system	A fuzzy logic (FL) control runs in the system, using instantaneous BP as the input variable governing the ultrafiltration rate (UFR) according to the BP trend	Prospective multicenter study<br><br>55 HD hypotension-prone HD patients	A fuzzy logic (FL) control ran in the system, using instantaneous BP as the input variable governing the ultrafiltration rate (UFR) according to the BP trend.<br><br>The system was user-friendly and just required the input of two data: critical BP (individually defined as the possible level of DH risk) and the highest UFR applicable (percentage of the mean UFR).<br><br>Sessions with (treatment A) and without (treatment B) ABPS were alternated for 30 dialysis sessions per patient (674 with ABPS vs 698 without).<br><br>Severe dialysis hypotension (DH) appeared in 8.3% of sessions in treatment A vs 13.8% in treatment B (-39%, p=0.01).

					Mild DH fell non-significantly (-12.3%). There was a similar percentage of sessions in which the planned body weight loss was not achieved and dialysis time was prolonged.<br><br>FL may be suited for interpreting and controlling the trend of a determined multi-variable parameter like BP.
Gaweda, A. E.	2008	Application of FL to predicting erythropoietic response in HD patients	A fuzzy logic (FL) sets were used in a pharmacodynamics model to represent the uncertainty about the classification of ESRD patient's response to EPO	Observational study<br><br>186 HD patients with anemia	The fuzzy categories contributed to a significant improvement in the classification of subjects with poor or normal response to erythropoietin.
Gaweda, A. E.	2008	Model predictive control of EPO administration in the anemia of ESRD	Model predictive control (MPC) by using an ANN model for evaluating the Hb response to ESA (erythropoietin stimulating agents).<br><br>Computer simulation to test MPC versus a conventional anemia management protocol (AMP)	Observational study<br><br>186 long-term HD patients	The achieved Hb levels were 12.3 +/- 0.6 g/dL for AMP and 11.6 +/- 0.4 g/dL for MPC ($p < 0.001$), mean SDs were 0.75 +/- 0.30 g/dL for AMP and 0.60 +/- 0.21 g/dL for MPC ($p < 0.01$), and mean absolute differences from target were 0.8 +/- 0.6 g/dL for AMP and 0.3 +/- 0.3 g/dL for MPC ($p < 0.001$).<br><br>MPC of ESAs (erythropoiesis-stimulating agents) may result in improved anemia management
Bhan, I.	2010	Clinical measures identify vitamin D deficiency in dialysis	ANNs, and decision trees with vitamin D deficiency as the dependent variable	Cohort study<br><br>10,044 incident HD patients	Accelerated Mortality on Renal Replacement (ArMORR) cohort of incident U.S. dialysis patients were divided into training (60%) and validation (40%) sets.<br><br>Black race, female sex, winter season, and hypoalbuminemia (serum albumin ≤3.1 g/dl) were the strongest predictors of vitamin D deficiency.<br><br>In the validation set, the presence of hypoalbuminemia and winter season increased the likelihood of vitamin D deficiency in black women (from 90% to 100%), black men (from 85% to 100%),

					white women (from 82% to 94%), and white men (from 66% to 92%).
Cadena, M.	2010	Method to observe hemodynamic and metabolic changes during hemodiafiltration therapy with exercise	BTM (Blood Temperature Monitor from Fresenius Inc), in order to determine how the dialysate temperature was controlled	Observational study<br><br>5 HD patients	A new method was used to observe the main physiological parameters: hearth rate variability (HRV), blood pressure, BTM dialysate temperature control and substrate utilization by indirect calorimtery, which were involved in hemodiafitration (HDF).<br><br>The results showed important advantages of this method which place the BTM performance as unstable control system with the possibility to produce undesirable HRV changes as the vagotonic response.
Jacob, A. N.	2010	ANNs analysis to predict mortality in ESRD: application to United States Renal Data System	Linear and neural network models for the accurately predicted survival in a dataset containing censored and uncensored patients	Observational study<br><br>242,576 patients who had an actual date of death defined within the United States Renal Data System (USRDS)	The average C statistic over a 6-month to 10-year time range achieved with these models was approximately 0.7891 (linear model), 0.7804 (transformed dataset linear model), 0.7769 (neural network model), 0.7774 (transformed dataset neural network model), 0.8019 (Cox model), and 0.7970 (transformed dataset Cox model).<br><br>These results suggested that data provided to the USRDS can allow for predictive models which have a high degree of accuracy years following the initiation of dialysis.
Azar, A. T.	2011	ANN for prediction of equilibrated dialysis dose without intradialytic sample	ANNs to predict the equilibrated urea (C eq) at 60 min after the end of HD	Observational study<br><br>150 HD patients	The mean urea rebound observed from the ANN was 18.6 ± 13.9%, while the means were 24.8 ± 14.1% and 21.3 ± 3.49% using Smye and Daugirdas methods, respectively.<br><br>The ANN model achieved a correlation coefficient of 0.97 (P <0.0001), while the Smye and Daugirdas methods yielded R = 0.81 and 0.93, respectively (P <0.0001); the errors of the Smye method were more significant than those of the other methods and resulted in a considerable bias in all cases, while the predictive accuracy for (eq Kt/V) 60 was equally good by the Daugirdas' formula and the ANN.<br><br>The use of the ANN urea estimation yielded accurate results when used to calculate (eq Kt/V).
Fuertinger, D. H.	2013	A Parameter Identification Technique for Structured Population Equations	A model for erythropoiesis, consisting of coupled	Not available	The numerical approximation for the population equations was based on semigroup theory, respectively on the theory of abstract Cauchy problems.

		Modeling Erythropoiesis in Dialysis Patients	partial differential equations, was adapted to an individual patient. A standard Least-Squares formulation was used to define the cost-functional used for parameter identification.		The system state was approximated by system states of high order differential equations on finite dimensional subspaces of the state space of the original system.<br><br>A low approximation dimension was sufficient for obtaining accurate numerical solutions and estimates for the parameters.
Titapiccolo, J. I.	2013	AI models to stratify cardiovascular risk in incident HD patients	Forecast models to predict the cardiovascular outcome of incident HD patients	Observational study<br><br>4246 incident HD patients	Random forest indicated higher performance with AUC of the ROC curve and sensitivity higher than 70% in both temporal windows models, proving that random forests were able to exploit non-linear patterns retrieved in the feature space.<br><br>Out-of-bag estimates of variable importance and regression coefficients were used to gain insight into the models implemented.<br><br>Malnutrition and an inflammatory condition strongly influenced cardiovascular outcome in incident HD patients.<br><br>The most important variables in the model were blood test variables such as the total protein content, percentage value of albumin, total protein content, creatinine and C reactive protein. Also, the age of patients and weight loss in the first six months of renal replacement therapy were highly involved in the prediction.<br><br>Among the built models random forests showed the best predictive performance.
Chen, W. L.	2014	A rule-based decision-making diagnosis system to evaluate arteriovenous shunt stenosis for HD treatment of patients using fuzzy petri nets	Rule-based decision-making diagnosis system evaluated arteriovenous shunt (AVS) stenosis for long-term HD treatment of patients using fuzzy petri nets (FPNs)	Observational study<br><br>42 HD patients	The Burg autoregressive (AR) method was used to estimate the frequency spectra of a phonoangiographic signal and identify the characteristic frequencies.<br><br>A rule-based decision-making method, FPNs, was designed as a decision-making system to evaluate the degree of stenosis (DOS) in routine examinations.<br><br>The examination results indicated that the proposed diagnosis system had greater efficiency in evaluating AVS stenosis.

Escandell-Montero, P.	2014	Optimization of anemia treatment in HD patients via reinforcement learning	Methodology based on reinforcement learning (RL) to optimize ESA therapy.<br><br>RL were formulated as Markov decision processes (MDPs)	Observational study<br><br>5000 HD patients	Simulation results showed that the performance of Q-learning was lower than fitted Q iteration (FQI) and the protocol.<br><br>FQI achieved an increment of 27.6% in the proportion of patients that were within the targeted range of hemoglobin during the period of treatment.<br><br>The quantity of drug needed was reduced by 5.13%, which indicated a more efficient use of ESAs.<br><br>The RL algorithm employed in the proposed methodology was fitted Q iteration, which stood out for its ability to make an efficient use of data. RL could represent an alternative to current protocols.
Nigwekar, S. U.	2014	Quantifying a rare disease in administrative data: the example of calciphylaxis	Algorithm to accurately identify cases of calciphylaxis and to examine its incidence and mortality	Observational study<br><br>Partners Research Patient Data Registry (RPDR): 11,451 chronic HD patients	The novel research strategy compared against the gold standard yielded indicated: sensitivity 89.2 %, specificity 99.9 %, positive likelihood ratio 3,382.3, negative likelihood ratio 0.11, and area under the curve 0.96.<br><br>Mortality rates among calciphylaxis patients were noted to be 2.5-3 times higher than average mortality rates for chronic HD patients.<br><br>By developing and successfully applying this novel algorithm a significant increase in calciphylaxis incidence was identified.
Barbieri, C.	2015	A new ML approach for predicting the response to anemia treatment in a large cohort of ESRD patients undergoing dialysis	Machine Learning (ML) to derive a predictor of the response to the ESA/Iron therapy in HD patients affected by secondary anemia	Retrospective observational study<br><br>688 patients from Italian clinics, 1397 patients from Spanish clinics and 2050 patients from Portuguese clinics	In CKD patients, Machine Learning (ML) was explicitly taken into account in order to produce a general and reliable model for the prediction of ESA/Iron therapy response.<br><br>The ML model makes use of both human physiology and drug pharmacology, yielding Mean Absolute Errors (MAE) of the Hemoglobin (Hb) prediction around or lower than 0.6 g/dl.<br><br>ML had predicted improvement based on red blood cell dynamics and drug kinetics.<br><br>ML improved the anemia management in dialysis and was suitable for the application in daily clinical practice.
Barbieri, C.	2016	Performance of a Predictive Model for Long-Term Hemoglobin Response to Darbepoetin and Iron	ANN algorithm for predicting hemoglobin concentrations three months into the future	Retrospective observational study	The model was able to predict individual variation of hemoglobin concentration 3 months in the future with a Mean Absolute Error (MAE) of 0.75 g/dL

		Administration in a Large Cohort of HD Patients		1558 HD patients treated with iv darbepoetin alfa and iv iron	The ANN predictive model offered a simple and reliable tool applicable in daily clinical practice for predicting the long-term response to ESA/iron therapy of HD patients.
Barbieri, C.	2016	An international observational study suggests that AI for clinical decision support optimizes anemia management in HD patients	Anemia Control Model (ACM) evaluated the impact of an AI decision support system on anemia outcomes	Retrospective observational study<br><br>752 HD patients	ACM introduction led to a significant decrease in hemoglobin fluctuation (intrapatient standard deviation decreased from 0.95 g/dl to 0.83 g/dl).<br><br>ACM support helped improve anemia outcomes of HD patients, minimizing erythropoietic-stimulating agent use with the potential to reduce the cost of treatment.
Brier, M. E.	2016	AI for optimal anemia management in end-stage renal disease	Computational intelligence	Not available	The models used for the prediction resulted from the use of individual patient data and helped increase the number of hemoglobin observations within the target range.<br><br>The benefits of using these modeling techniques appeared to be a decrease in erythropoiesis-stimulating agent use and a decrease in the number of transfusions.
Rodriguez, M.	2016	A New Data Analysis System to Quantify Associations between Biochemical Parameters of CKD-Mineral Bone Disease	RF (Random Forest) used to quantify the degree of association between parameters of chronic kidney disease and mineral bone disease (CKD-MBD)	Cohort study<br><br>1758 adult HD patients	RF assumed that changes in phosphate would cause modifications in other associated variables (calcium and others) that may also affect PTH values.<br><br>The analysis revealed a strong association between PTH and phosphate that was superior to that of PTH and Calcium.<br><br>RF assumed that changes in phosphate would cause modifications in other associated variables (calcium and others) that may also affect PTH values.<br><br>Using RF the correlation coefficient between changes in serum PTH and phosphate was 0.77, $p<0.001$; thus, the power of prediction markedly increased. The effect of therapy on biochemical variables was also analyzed using this RF.
Saadat S	2017	Predicting Quality of Life Changes in HD Patients Using ML: Generation of an Early Warning System	ML (machine learning) used to predict changes in the quality of life scores of HD patients for the subsequent month and the	Prospective cohort study<br><br>78 HD patients	Using ML algorithms, two models (classification tree and Naïve Bayes) were generated to predict an increase or decrease of 5% in a patient's World Health Organization Quality Of Life-BREF (WHOQOL-BREF) score over one month.

			development of an early warning system		The classification tree was selected as the most accurate model with an area under curve (AUC) of 83.3% (accuracy: 81.9%) for the prediction of 5% increase in quality of life (QOL) and an AUC of 76.2% (accuracy: 81.8%) for the prediction of 5% decrease in QOL over the coming month.<br><br>The factors associated with an increase of QOL by 5% or more over the next month included younger age (<19 years) and higher iron sucrose doses (>278mg/month).<br><br>An early warning system, dialysis data interpretation for algorithmic-prediction on QoL was built for the early detection of deteriorating QOL scores in the HD population using ML algorithms.
Bhatia, G.	2018	ML for Prediction of Life of Arteriovenous Fistula	ML for Prediction of Life of Arteriovenous Fistula	Observational study<br><br>200 HD patients	This ML provided similar data points to those required for the health of the fistula and proposed a mechanism to predict the life of a fistula
Brier, M. E.	2018	Personalized Anemia Management and Precision Medicine in ESA and Iron Pharmacology in ESRD	ANNs, physiologic models, and feedback control systems to provide erythropoietic dosing information for patients with anemia resulting from chronic kidney disease	Review<br><br>Not available	All methods indicated an advantage in at least one area over the traditional paper expert system used by most dialysis facilities.<br><br>This study showed improvements in the percentage of hemoglobin measurements within target range, decreased within-subject hemoglobin variability, decreased erythropoiesis-stimulating agent dose, and decreased transfusion rates.
Bucalo, M. L.	2018	The anaemia control model: Does it help nephrologists in therapeutic decision-making in the management of anaemia?	Anemia control model (ACM) to assess the usefulness in the treatment of anemia in HD	Retrospective multinational study<br><br>213 HD patients in the intervention phase 1, 219 in the control period and 218 in the intervention phase 2	ACM was a software that predicted the optimal dose of darbepoetin and iron sucrose to achieve target hemoglobin (Hb) and ferritin levels, and made prescription suggestions.<br><br>ACM increased the percentage of Hb in range: 80.9% in intervention phases (IP2), compared with 72.7% in the control phase (CP).<br><br>ACM reduced the intake of darbepoetin (IP1: 20 [70]; CP 30 [80]µg, p=0.032) with less Hb fluctuation (0.91±0.49 in the CP to 0.82±0.37g/dl in IP2, p<0.05), improving in the ACM compliant group.

					ACM helped obtain better anemia results in HD patients, minimizing the risks of treatment with ESAs and reducing costs.
Chao, P. C.	2018	A Portable, Wireless Photoplethysomography Sensor for Assessing Health of Arteriovenous Fistula Using Class-Weighted Support Vector Machine	Wireless photoplethysomography (PPG) sensor for assessing arteriovenous fistula (AVF)	Observational study<br><br>5 HD patients: 3 patients with healthy AVF and 2 patients with dysfunctional AVF	The proposed PPG sensors successfully achieved an accuracy of 89.11% in assessing thev health of AVF and with a type II error of 9.59%.<br><br>A class-weighted SVM (Support Vector Machine) classifier was proposed for assessing the health of AVF.
Niel O.	2018	AI outperforms experienced nephrologists to assess dry weight in pediatric patients on chronic hemodialysis.	ANN to improve the accuracy of dry weight assessment in HD patients	Observational study<br><br>14 pediatric patients	Artificial intelligence dry weight was higher (28.6%), lower (50%), or identical to nephrologist dry weight.<br><br>Mean difference between artificial intelligence and nephrologist dry weights was 0.497 kg (- 1.33 to + 1.29 kg).<br><br>In patients for whom artificial intelligence dry weight was lower than nephrologist dry weight, systolic blood pressure significantly decreased after dry weight decrease to artificial intelligence dry weight (77th to 60th percentile, p = 0.022); anti-hypertensive treatments were successfully decreased or discontinued in 28.7% of cases.<br><br>In patients for whom artificial intelligence dry weight was higher than nephrologist dry weight, no hypertension was observed after dry weight increase to artificial intelligence dry weight.<br><br>Neural network predictions outperformed those of experienced nephrologists in most cases, proving that artificial intelligence was a powerful tool for predicting dry weight in HD patients.
Usvyat, L.	2018	Using Technology to Inform and Deliver Precise Personalized Care to Patients With ESRD	Health care models and ML to deliver precise personalized care to HD patients	Review<br><br>Not available	Data from health care and non-health care sources and advanced analytical methods such as ML can be used to create novel insights, and large volumes of data can be integrated to support clinical decisions.<br><br>Health care models continue to evolve and the opportunities and need for novel care approaches supported by technology and health

					informatics continue to expand as the delivery and organization of health care changes.
Barbieri, C.	2019	Development of an AI Model to Guide the Management of Blood Pressure, Fluid Volume, and Dialysis Dose in ESRD Patients: Proof of Concept and First Clinical Assessment	Multiple-endpoint model predicting HD session<br><br>ANN	Observational study<br><br>2 HD patients	State-of-the-art AI was adopted in other complex decision-making tasks for dialysis patients and may help personalize the multiple dialysis-related prescriptions affecting patients' intradialytic hemodynamics.<br><br>A multiple-endpoint predicted session-specific Kt/V, fluid volume removal, heart rate, and BP based on patient characteristics, historic hemodynamic responses, as well as dialysis-related prescriptions.<br><br>The accuracy and precision of this preliminary model was encouraging and may be used to anticipate patients' reactions through simulation so that the best strategy can be chosen based on clinical judgment or formal utility functions.
Hueso, M.	2019	Progress in the Development and Challenges for the Use of Artificial Kidneys and Wearable Dialysis Devices	Novel wearable dialysis devices<br><br>ANN	Review<br><br>Not available	The development of novel wearable dialysis devices and the improvement of clinical tolerance will need contributions from new branches of engineering such as AI and ML for the real-time analysis of equipment alarms, dialysis parameters, and patient-related data with a real-time feedback response.<br><br>Emerging technologies derived from AI, ML, electronics, and robotics may offer great opportunities for dialysis therapy, but much innovation is needed before we achieve a smart dialysis machine able to analyze and understand changes in patient homeostasis and to respond appropriately in real time.

Supplemental Table 2.

Author	Year	Title of study	Type of AI/ML algorithm used	Type of study & No PD patients	Results
Zhang, M.	2005	Selection of PD schemes based on multi-objective fuzzy pattern recognition	Multi-objective fuzzy pattern recognition and its application in the selection of PD schemes	Not available	The method was first applied to the field of PD.<br><br>The conclusion showed that this method is compliant with doctors' opinions. It provided a new idea for research in this field. At the same time, since the method was simple and easy to use, it can be of wide application.
Chen, C. A.	2006	Neural network modeling to stratify peritoneal membrane transporter in predialytic patients	ANN model for predialytic stratification of uremic patients on the basis of peritoneal membrane transport status.	Observational study<br><br>111 PD patients	The ANN model demonstrated the usefulness of the model to stratify predialytic patients into H (high average transporters) and L (low average transporters) groups by its significant discrimination (AUC=0.812>0.7) and best fitted calibration (p value of H-statistic=0.421>0.05).<br><br>The evaluation of peritoneal membrane transport status helped clinicians offer their uremic patients better therapeutic options.<br><br>The ANN model appeared to be a promising tool for stratifying predialytic patients on the basis of peritoneal membrane transport status and helped clinicians make decisions about which dialysis modality was suitable.
Tangri, N.	2011	Determining factors that predict technique survival on PD: application of regression and ANNs methods	ANNs and logistic and Cox regression methods used to compare the factors that predict technique survival	Prospective study collected data from the United Kingdom Renal Registry<br><br>3269 PD patients	Removal of dialysis center code had a significant effect on the fit and/or predictive performance of all three types of models.<br><br>The effect of demographic data, comorbidity, physical examination and laboratory data varied according to the type of model.<br>Other putative predictive factors had marginal and/or variable effects.
Rodrigues, M.	2017	Understanding Stroke in Dialysis and CKD	Data Mining Models	Observational study	These patterns can be used by decision support systems to determine diagnoses, prognoses and treatments for patients in healthcare organizations and present them as aids to physicians.

			CRISP-DM (Cross Industry Standard Process for Data Mining): Business Understanding, Data Understanding, Data Preparation, Modelling and Evaluation and Deployment<br><br>NaiveBayes (NB), Logistics Regression (LR), Multilayer Perceptron (MP), Random Tree (RT) and K-neighbors (IBK)	86 PD patients with stroke risk	The threshold created was 95% Sensitivity, 95% Specificity and 95% Accuracy.<br><br>RT and IBK had the best results compared to all the other algorithms mentioned specially because they were the only algorithms whose Sensitivity, Specificity and Accuracy were higher than 95%.<br><br>Using this algorithm would provide the medical staff with a high quality with a low error informed diagnosis.
Zhang, J.	2017	ML algorithms define pathogen-specific local immune fingerprints in PD patients with bacterial infections	ML to characterize responses to microbiologically well-defined infection	Cohort study<br><br>83 PD patients on the day of presentation with acute peritonitis	The ML algorithm demonstrated that different groups of bacteria induced qualitatively distinct local immune fingerprints, with specific biomarker signatures associated with Gram-negative and Gram-positive organisms, and with culture-negative episodes of unclear etiology.<br><br>This method had diagnostic and prognostic implications by informing patient management and treatment choice at the point of care.<br><br>This method established the power of non-linear mathematical models to analyze complex biomedical datasets and highlight key pathways involved in pathogen-specific immune responses.
Fernandez-Lozano, C.	2018	A generalized linear model for cardiovascular complications prediction in PD patients	Four different machine learning techniques: s RF, SVM, GLM- NET and KNN to identify patient non-invasive information for cardiovascular complications prediction in PD patients	Observational study<br><br>114 PD patients	The best classification algorithm was a Generalized Linear Model, which achieved AUC values above 96% using a small subset of the original variables following a feature selection approach.<br><br>This approach allowed to increase the interpretability of the combinations of traditional factors, advanced CKD factors and PD factors, all related with a cardiovascular risk profile.
Brito, C.	2019	A DM approach to classify serum creatinine values in	Data mining (DM) helped in classifying the values of serum creatinine in	Observational study	The classification process can find patterns useful to understand the patients' health development and to perform the medical act according to such results.

		patients undergoing continuous ambulatory PD	patients undergoing continuous ambulatory peritoneal dialysis (CAPD)<br><br>The group of algorithms included: the IBK algorithm, the KStar algorithm, Reduced Error Pruning (REP) Tree, Random Forest algorithm, The Random Tree algorithm	2489 medical examinations of different patients	DM was a highly satisfactory method, reaching accuracy rate values of approximately 95%, and low relative absolute error values.
John, O.	2019	Remote Patient Management in PD: An Answer to an Unmet Clinical Need	Remote patient management (RPM) to improve health outcomes and simulate a real-time clinician-patient interaction	Review<br><br>Not available	Remote patient management (RPM) had the potential to improve outcomes in PD through telehealth platforms that facilitated virtual clinical presence.<br><br>RPM enabled patient-generated clinical documentation and feedback mechanism and promoted self-monitoring.<br><br>RPM enabled the clinicians to closely monitor and detect early issues, provide feedback in real-time, and initiate early interventions prescription modifications and contextual clinical decision support.<br><br>ML and AI algorithms would help detect patterns and predict impending complications (*e.g.* luid overload, heart failure or peritonitis), allowing early detection to avoid hospitalizations.

Supplemental Table 3.

Author	Year	Title of study	Type of AI/ML algorithm used	Type of study & No KT patients	Results
Simic-Ogrizovic, S.	1999	Using ANN in selection of the most important variables in prediction of chronic renal allograft rejection progression	ANNs (artificial neural networks) can be useful in the evaluation of chronic renal allograft rejection progression	Retrospective study<br><br>27 patients with a clinical and pathologic (Banff classification) confirmed CR diagnosis	ANN identified a correlation between the selected variables and reduced the initial set of 33 variables to a set of several variables, which were best correlated with chronic rejection (CR) progression.<br><br>This method provided the same validity of the identification, although the number of features was reduced. ANN seemed more reliable in the prediction of the CR course than the usual statistical methods.
Fritsche, L.	2002	Recognition of critical situations from time series of laboratory results by case-based reasoning	A case-based reasoning algorithm using *dynamic time-warping* for kidney transplant recipients, for acute rejections<br><br>ANN	Observational study<br><br>1,143 patients and 680 rejection episodes	The accuracy of the algorithm increased steadily with the size of the available case base. With the largest case bases, the case-based algorithm reached an accuracy of 78 +/- 2%, which was significantly higher than the performance of experienced physicians (69 +/- 5.3%) ($p < 0.001$).<br><br>The new case-based reasoning algorithm with dynamic time warping as the measure of similarity allowed the extension of the use of automatic laboratory alerting systems to conditions in which abnormal laboratory results were the norm and critical states could be detected only by recognition of pathological changes over time.
Stachowska, E.	2006	The use of ANNs in evaluation of the direction and dynamics of changes in lipid parameters in kidney transplant patients on the Mediterranean diet	ANNs can be a tool useful in the evaluation of the effect of the Mediterranean diet (MD)	Randomized, prospective study<br><br>21 patients after kidney transplantation<br><br>16 patients (after transplantation) on a low-fat diet, isocaloric	The MD diet would be ideal for posttransplantation patients without serious pathologic dyslipidemia. In the case of patients with substantial dyslipidemia, appropriate pharmacologic treatment lowering proatherosclerotic lipid levels should be used in combination with the MD.<br><br>Artificial neural networks (ANNs) were a useful tool in modeling biological parameters, showing the dynamics of the studied interactions in a very detailed way. ANN was the most suitable method for investigations with many variables, interconnected nonlinearly; it also allowed for a more general approach to biological problems.<br><br>To ensure the predictive power of this method for new cases, the representative database was indispensable, and ANN proved to

					be a prospective tool for reliable, quick assessments and predictions.
Santori, G.	2007	Application of an ANN model to predict delayed decrease of serum creatinine in pediatric patients after kidney transplantation	Effectiveness of ANN model to predict a delayed decrease of serum creatinine	Retrospective study<br><br>107 pediatric kidney recipients	The neural network showed sensitivity and specificity for the whole patient cohort were 0.875 and 0.87, respectively, whereas using logistic regression sensitivity and specificity yielded 0.37 and 0.94, respectively.<br><br>The neural network model seemed to predict a delayed of decrease in serum creatinine among pediatric kidney recipients.<br><br>The availability of the source code may allow the development of stand-alone neural networks to validate this model in prospective studies.
Sharma, D.	2008	An intelligent multi-agent design in healthcare management system	The multi-agent approach for the development of complex e-health systems	Not available	The use of intelligent multi-agent approach was important for developing e-health systems, for the prediction of kidney transplant outcomes and the management of chronic diseases such as diabetes.<br><br>The kidney transplant outcome prediction was based on the use of a novel classification approach which was a combination of initial data preparations, preliminary classification by ensembles of neural networks, generation of new training data based on criteria of highly accuracy and model agreement, and decision trees.
Greco, R.	2010	Decisional trees in renal transplant follow-up	Decision trees were widely used to represent classification rules in a population by a hierarchical sequential structure	Retrospective study<br><br>194 renal transplant patients with 5 years of follow-up	The classification algorithm produced a decision tree that allowed to evaluate the interactions between ARE (acute rejection episode), DGF (delayed graft function), CAN (chronic allograft nephropathy), and BMI on graft outcomes, producing a validation set with 88.2% sensitivity and 73.8% specificity.<br><br>This model was able to highlight that subjects at risk of graft loss experienced one or more events of ARE, developed DGF and CAN, or had a BMI > 24.8 kg/m2 and CAN.<br><br>The use of decision trees in clinical practice may be a suitable alternative to the traditional statistical methods, since it may allow one to analyze interactions between various risk factors beyond the previous knowledge.

Brown, T. S.	2012	Bayesian modeling of pretransplant variables accurately predicts kidney graft survival	Bayesian Belief Network (BBN) to determine whether a predictive model of graft survival can be derived using pre-transplant variables	Observational study<br><br>5,144 randomly selected patients from the United States Renal Data System database	BBN model was able to predict graft failure within the first year or within 3 years (sensitivity 40%; specificity 80%; area under the curve, AUC, 0.63).<br><br>Recipient BMI, gender, race, and donor age were amongst the pre-transplant variables with strongest association to outcome.<br>A 10-fold internal cross-validation showed similar results for 1-year (sensitivity 24%; specificity 80%; AUC 0.59) and 3-year (sensitivity 31%; specificity 80%; AUC 0.60) graft failure.<br><br>BBN enabled to examine variables from a large database to develop a robust predictive model.
Seeling, W.	2012	Knowledge-based tacrolimus therapy for kidney transplant patients	Conditional Inference Trees (CITs) estimated statistically the Tacrolimus blood level thresholds.	Observational study<br><br>492 KT patients	The CIT formed homogenous classes to eliminate the confounding effect.<br><br>CIT was the first approach of more accurate forecasting models for the medication blood level of the respective medication.<br><br>The next step could be to add weights, data of rejections or other covariates, which may be able to improve the model even further, but this data was not easily accessible.
Decruyenaere, A.	2015	Prediction of delayed graft function after kidney transplantation: comparison between logistic regression and ML methods	ML methods in the prediction of delayed graft function (DGF).	Cohort study<br><br>497 KT patients	The discriminative capacities of LDA (linear discriminant analysis), linear SVM, radial SVM (support vector machines) and LR (logistic regression) were the only ones above 80%.<br><br>None of the pairwise AUROC comparisons between these models were statistically significant, except linear SVM outperforming LR.<br><br>The sensitivity of linear SVM to identify recipients with DGF was amongst the three highest of all models, while SVM was the most appropriate to predict DGF.
Karademirci, O.	2015	Implementation of a User-Friendly, Flexible Expert System for Selecting Optimal Set of Kidney Exchange Combinations of Patients in a Transplantation Center	NP-Hard (Non-deterministic Polynomial-time hard) combinatorial to settle the kidney exchange problem	Observational study<br><br>60 patients in the kidney exchange pool	The patient donor information in the Excel spreadsheets was processed by a code developed in Visual Basic (VB) language used for Excel macros. The VB code generated all possible 2-wise and 3-wise transplantation combinations between patients and donor based on their information (blood group compatibility, age compatibility, etc).

			Decision support to use in transplantation centers, facilitating their operations. system		A mathematical model developed in Cplex mathematical modeling language used data prepared by VB code and calculated the optimal selection of the transplantation combinations.<br><br>Cplex code output the results in the same spreadsheet organ transplantation coordinators used.<br><br>The decision support system could be modified according to the matching preferences of transplantation centers and it could be used as a simulation tool for analyzing different allocation methods.
Srinivas	2017	Big Data, Predictive Analytics, and Quality Improvement in Kidney Transplantation: A Proof of Concept	Big Data Solution to predict graft loss (GL) and mortality	Single - center retrospective cohort<br><br>891 adult solitary kidney transplant recipients transplanted	Model 1: United Network for Organ Sharing (UNOS) data; Model 2: UNOS & Transplant Database (Tx Database) data; Model 3: UNOS, Tx Database & EHR comorbidity data; and Model 4: UNOS, Tx Database, EHR data, Posttransplant trajectory data, and unstructured data. A 10% 3-year GL rate was observed among patients.<br><br>Layering of data sources improved model performance; Model 1: area under the curve (AUC), 0.66; (95% confidence interval [CI]: 0.60, 0.72); Model 2: AUC, 0.68; (95% CI: 0.61-0.74); Model 3: AUC, 0.72; (95% CI: 0.66-077); Model 4: AUC, 0.84, (95 % CI: 0.79-0.89). One-year GL (AUC, 0.87; Model 4) and 3-year mortality (AUC, 0.84; Model 4) models performed similarly.
Tang, J.	2017	Application of Machine-Learning Models to Predict Tacrolimus Stable Dose in Renal Transplant Recipients	Eight machine learning techniques in pharmacogenetic algorithm-based prediction of Tacrolimus stable dose (TSD):<br>MLR, ANN, regression tree (RT), multivariate adaptive regression splines (MARS), boosted regression tree (BRT), support vector regression (SVR), random forest	Cohort study<br><br>1,045 renal transplant patients	Generally, the RT algorithm provided more accurate prediction of TSD than the other 8 algorithms.<br><br>Patient doses in the intermediate range were best predicted compared with the actual stable dose in both the derivation and the validation cohorts (MAE = 0.50 and 0.48 mg/day, respectively) (Table 2). For patients who required 2.5 mg/day or less (24.4% of a total of 693 patients), 38.5% of the predicted dosage fell into ideal dose range (20% of the actual dose). While for the patients who required 4 mg/day or more (20.8% of the total number of patients), 44.4% of the prediction dropped into the ideal dose.<br><br>Among all the ML models, RT performed best in both the derivation [0.71 (0.67–0.76)] and the validation cohorts [0.73

			regression (RFR), lasso regression (LAR) and Bayesian additive regression trees (BART)		(0.63–0.82)]. In addition, the ideal rate of RT was 4% higher than that of MLR.
Yoo, KD.	2017	A ML Approach Using Survival Statistics to Predict Graft Survival in Kidney Transplant Recipients: A Multicenter Cohort Study	Learning algorithms (survival decision tree, bagging, RF and ridge and lasso) for accurate prediction of graft survival after kidney transplant	Multicenter cohort study<br><br>3,117 KT recipients	The accurate prediction of graft survival after kidney transplant was limited by the complexity and heterogeneity of risk factors influencing allograft survival.<br><br>In this study, ML methods were applied in combination with survival statistics, to build new prediction models of graft survival that included immunological factors, as well as known recipient and donor variables.<br><br>Machine learning methods may provide versatile and feasible tools for forecasting graft survival.
Gallon, L.	2018	Intragraft Molecular Pathways Associated with Tolerance Induction in Renal Transplantation	Prediction analysis showed inhibition of pro-inflammatory regulators and activation of anti-inflammatory pathways in FCRx samples	Pilot study<br><br>27 KT patients : FCRx-induced tolerant protocol (FCRx; *n*=7), diagnosed with rejection (R; *n*=10), and without acute rejection but under standard immunosuppression (SIS; *n*=10)	The integrative analyses (microRNA and gene expression profiling from the same biopsy sample) identified the induction of regulators with demonstrated roles in the downregulation of inflammatory pathways and maintenance of tissue homeostasis in tolerance-induced FCRx (bioengineered stem cell product) samples compared with SIS (standard immunosuppression) samples).<br><br>This study highlighted the utility of molecular intragraft evaluation of pathways related to FCRx-induced tolerance and the use of integrative analyses for identifying upstream regulators of the affected downstream molecular pathways.
Jia, L.	2018	LCK as a Potential Therapeutic Target for Acute Rejection after Kidney Transplantation: A Bioinformatics Clue	Gene Ontology (GO) functional enrichment analysis and Kyoto Encyclopedia of Genes and Genomes (KEGG) pathway analysis<br><br>Protein-protein interactions (PPIs) and literature mining were applied to construct the	Observational study<br><br>kidney biopsies from 30 stable renal allograft recipients, 15 patients with AR, 15 with BK virus nephropathy, and 12 with chronic allograft nephropathy	A total of 437 upregulated genes and 353 downregulated genes were selected according to $p<0.01$.<br>Differentially expressed genes (DEGs) of AR were mainly located on membranes and impacted the activation of receptors in immune responses.<br><br>In the PPI network, Src kinase, lymphocyte kinase (LCK), CD3G, B2M, interferon-γ, CD3D, tumor necrosis factor, VAV1, and CD3E in the T cell receptor signaling pathway were selected as important factors, and LCK was identified as the hub protein.

			co-citation network and to select the hub protein.<br><br>Bioinformatics methods to identify the key biomarker of acute rejection (AR) after kidney transplantation		LCK, via acting on T-cell receptor, might be a potential therapeutic target for AR after kidney transplantation.
Niel, O.	2018	AI improves estimation of tacrolimus area under the concentration over time curve in renal transplant recipients	ANNs to estimate of Tacrolimus AUC over time curve in KT	Not available	An ANN was implemented to approximate immediate release Tacrolimus AUC in renal transplant recipients. Neural network was a multi−layer perceptron network made of sigmoid neurons.<br><br>There was no significant difference between mean AUC and mean nnAUC values (187 and 188.6 ng h/ml, respectively, p= 0.95). AUC and nnAUC variances were not significantly different (p= 0.60).<br><br>AI seemed to be a fast, cheap, and clinically simple approach to estimate Tacrolimus AUC in renal transplant recipients.<br><br>Neural network required Tacrolimus blood concentration at 3 h postdose to compute nnAUC; this parameter was cheap and easy to obtain, especially in children or elderly patients. Neural network predictions of Tacrolimus exposure outperformed predictions of other models, including recent Bayesian models.
Rashidi Khazaee, P.	2018	A dynamic model for predicting graft function in kidney recipients' upcoming follow up visits: A clinical application of ANN	ANN based prediction models for predicting an eGFR value<br><br>Models' performances were evaluated by Mean Square Error (MSE) and Mean Absolute Error (MAE)	Cohort study<br><br>675 kidney recipients	The most accurate model included 3 static covariates of recipients' gender, donors' age and gender as well as 11 dynamic covariates of recipients including current age, time since transplant, serum creatinine, fasting blood sugar, weight and blood pressures available at each visit time.<br><br>The performance of prediction models in the validation cohort was improved when history window of time dependent variables' recent values was increased from 1 to 10 (an MSE decline from 161 to 99).<br><br>The best performed model was able to dynamically predict a future eGFR value for kidney recipients' upcoming visits.

					Integrating such a clinical tool into daily workflow of outpatient clinics could potentially support clinicians in optimal and individualized decision
Thishya, K.	2018	ANN model for predicting the bioavailability of tacrolimus in patients with renal transplantation	ANN and logistic regression (LR) models were used to predict the bioavailability of Tacrolimus and the risk for post-transplant diabetes	Prospective study<br><br>129 patients undergoing renal transplantation	The five-fold cross-validation of ANN model indicated good correlation with the experimental data of bioavailability (r2 = 0.93-0.96).<br><br>LR model showed an independent association of ABCB1 2677 G>T/A with post-transplant diabetes (OR: 4.83, 95% CI: 1.22-19.03).<br><br>Multifactor dimensionality reduction analysis (MDR) revealed that synergistic interactions between CYP3A5*3 and ABCB1 2677 G>T/A as the determinants of risk for post-transplant diabetes.<br><br>The ANN and MDR models explored both individual and synergistic effects of variables in modulating the bioavailability of Tacrolimus and the risk for post-transplant diabetes.
